# Clinical Outcome of Combined Types 1, 2, and 3 Lateral Hinge Fractures After Open-Wedge High Tibial Osteotomy: A Case Report

**DOI:** 10.7759/cureus.82863

**Published:** 2025-04-23

**Authors:** Nobuaki Hayashi, Manato Horii, Seiji Ohtori, Takahisa Sasho

**Affiliations:** 1 Department of Orthopedic Surgery, Graduate School of Medical and Pharmaceutical Sciences, Chiba University, Chiba, JPN; 2 Center for Preventive Medical Sciences, Chiba University, Chiba, JPN

**Keywords:** bone atrophy, lateral hinge fracture, low-intensity pulsed ultrasound, open wedge high tibial osteotomy, takeuchi classification

## Abstract

Lateral hinge fractures (LHFs) after open-wedge high tibial osteotomy (OWHTO) pose the risk of delayed bone union and loss of correction, and their management remains a challenge. The following case report describes a rare case of combined type 1, 2, and 3 LHFs following OWHTO. The patient was a 48-year-old female with a history of desmoid tumor resection and radiotherapy on the ipsilateral posterior aspect of the thigh, which was distal to the popliteal area of the knee. Bone atrophy was observed in the proximal tibia. OWHTO was performed for medial knee osteoarthritis (Kellgren-Lawrence grade 3) with a correction angle of 13°. Two months after surgery, types 1, 2, and 3 LHFs were identified. Treatment included weight-bearing restriction and low-intensity pulsed ultrasound (LIPUS), which resulted in successful bone healing. During the 16-month follow-up, the patient was pain-free and could walk smoothly. This case was complicated by types 1, 2, and 3 LHFs after OWHTO because of factors such as large preoperative hip-knee-ankle angle, proximal tibia bone atrophy, insufficient screw length, and the distance between the plate and bone. The combination of weight-bearing restriction and LIPUS effectively managed multiple LHFs after OWHTO, preventing significant correction loss and nonunion by promoting bone healing.

## Introduction

Open-wedge high tibial osteotomy (OWHTO) is an effective surgical method for treating medial knee osteoarthritis (OA) and has favorable short- to long-term clinical outcomes [1,2]. Lateral hinge fractures (LHFs), a critical complication of OWHTO, occur in 25-50% of cases and may cause limited physical activities in daily life [3-6]. Bone union tends to be substantially delayed when OWHTO is complicated by an LHF [7]. In some cases, the osteotomy site may develop nonunion, potentially requiring additional surgery [8]. LHFs were classified by Takeuchi et al. [3] into three types: type 1 fractures, in which the fracture line is within the proximal tibiofibular joint or up to its proximal lateral cortex; type 2 fractures, in which the fracture line is within the distal portion of the proximal tibiofibular joint; and type 3 fractures, in which the fracture line reaches the medial tibial joint surface. Type 1 fractures are stable, whereas type 2 and 3 fractures are unstable, with the risk of delayed bone union or loss of correction. However, no cases with multiple types of LHFs after OWHTO have been reported, and the clinical outcomes of the treatment of multiple types of LHFs are unknown. Here, we report the physical and radiographic characteristics and clinical outcomes of a case of combined types 1, 2, and 3 LHFs after OWHTO.

## Case presentation

Patient information

A 48-year-old female presented with right knee pain that was refractory to conservative treatments, including oral medications, intra-articular injections, and physical therapy. The patient had a history of surgical resection and radiation therapy for a desmoid tumor on the ipsilateral posterior aspect of the thigh, distal to the popliteal area of the knee, which had occurred 26 years prior to this event. The patient subsequently underwent ipsilateral Achilles tendon lengthening 21 years prior, owing to adhesive changes in the lower limb that complicated the aforementioned surgery. The patient had a history of obsessive-compulsive disorder but no history of smoking.

Clinical findings

Upon examination, the patient was found to be 152 cm tall and weighed 54 kg. Physical assessment revealed a positive patellar ballottement test result. The range of motion (ROM) of the right knee ranged from 3° in flexion to 135°. The patient experienced tenderness along the medial line of the joint. She did not exhibit lateral joint tenderness or knee joint instability. However, a large surgical scar extending from the right buttock to the proximal posterior lower leg was visible. Notably, knee extension was preserved with the hip in extension; however, hip flexion caused simultaneous knee flexion due to tension in the posterior medial musculature of her knee.

Imaging findings

**Figure 1:**
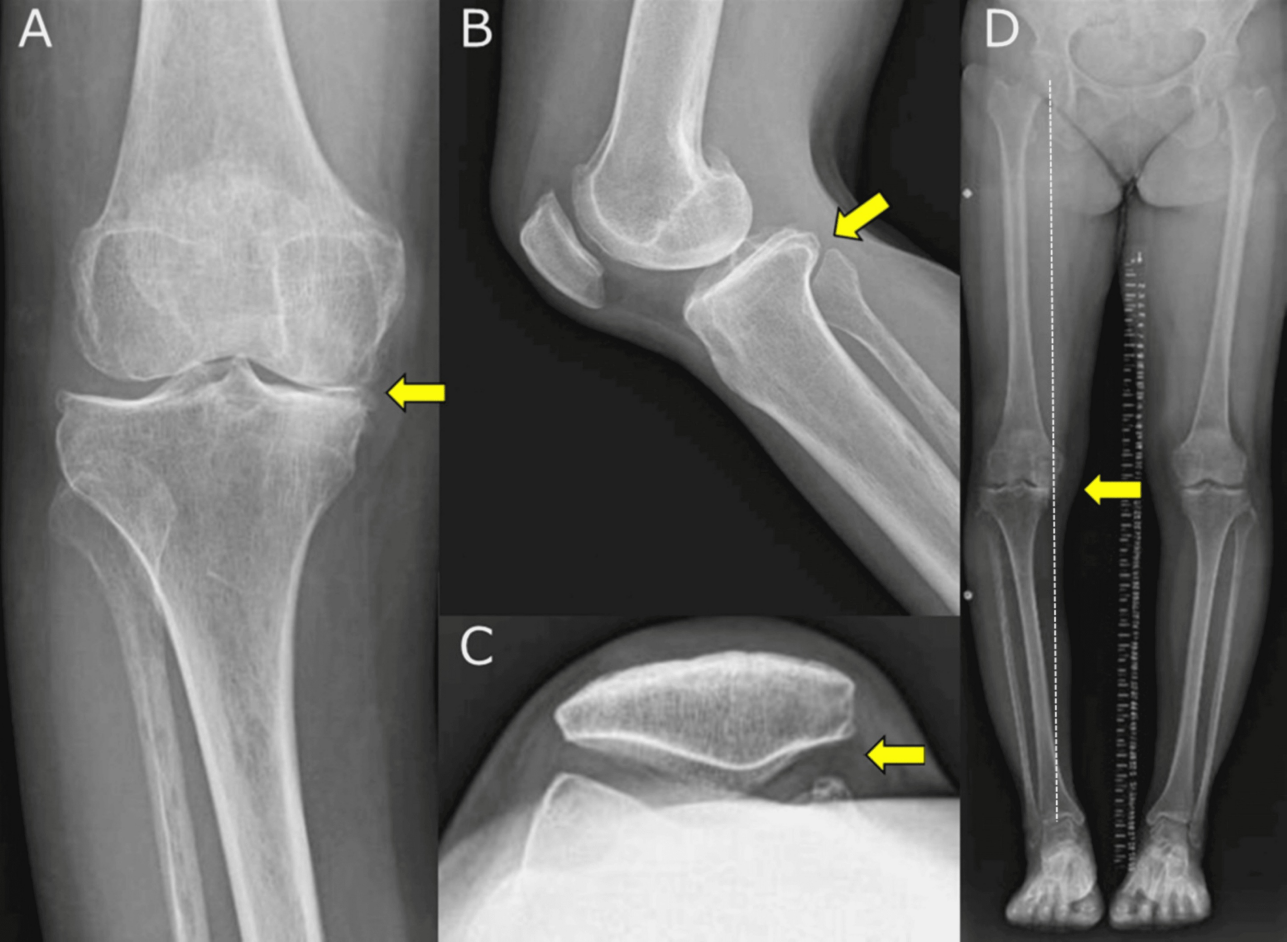
Preoperative plain radiograph of the knee (A) Frontal radiograph showing KL grade 3 OA in the medial compartment (yellow arrow), along with proximal tibial atrophy. (B) Lateral radiograph showing a posterior tibial slope (yellow arrow). (C) Skyline view radiograph showing no OA in the PF joint (yellow arrow). (D) Full-length lower extremity radiograph showing varus deformity of the lower extremity (yellow arrow). The white dotted line indicates the Mikulicz line. KL, Kellgren-Lawrence; OA, osteoarthritis; PF, patellofemoral

**Figure 2:**
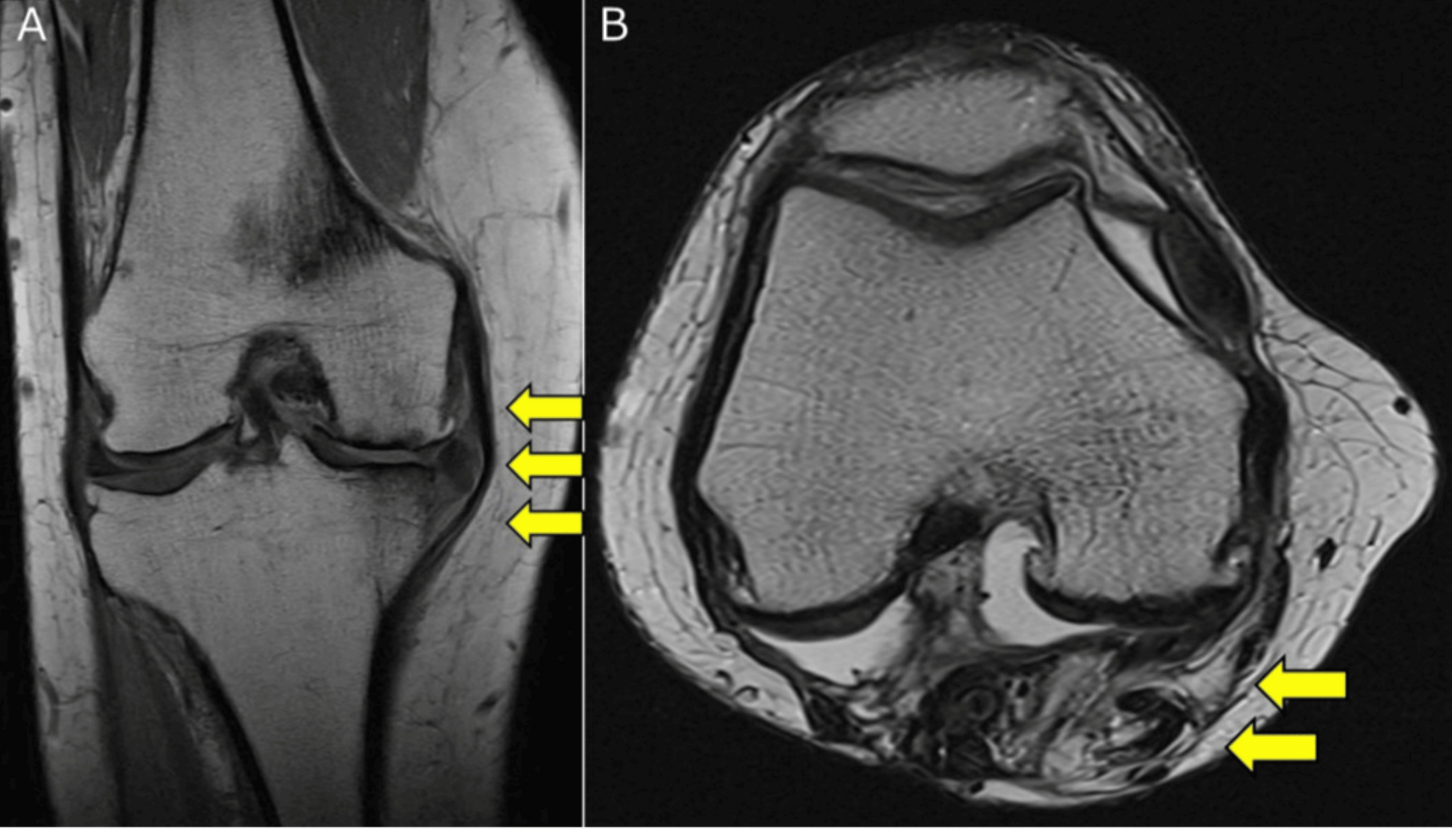
Preoperative proton density-weighted MRI scan of the right knee (A) Coronal image of the right knee showing medial meniscus extrusion and extensive cartilage degeneration in the medial FT joint (yellow arrows), with the lateral FT joint remaining intact. (B) Axial image of the right knee showing a surgical defect in the gastrocnemius muscle and a scar in the same area (yellow arrows). FT, femorotibial

Radiography revealed Kellgren-Lawrence grade 3 OA in the medial compartment of the knee, proximal tibial bone atrophy, and no OA in the patellofemoral (PF) joint. A full-length lower extremity radiograph showed the following alignment parameters: % mechanical axis (% MA) 0.0%, mechanical hip-knee-ankle (mHKA) angle 9.5°, anatomical femorotibial angle (aFTA) 184.5°, medial proximal tibial angle (MPTA) 83.2°, joint line convergence angle (JLCA) 6.4°, mechanical lateral distal femoral angle (mLDFA) 86.2°, and posterior tibial slope (PTS) 5.6° (Figure 1). MRI revealed medial meniscal extrusion and extensive cartilage degeneration in the medial femorotibial (FT) joint. In contrast, the cartilage in the lateral FT and PF joints and ligaments of the knee joints remained intact. Gastrocnemius muscle defects, due to extensive surgical removal, and a scar were observed in the same area (Figure 2).

Preoperative planning

Owing to the effects of surgery on the desmoid tumor, adhesions formed on the posteromedial side of the knee. To release these adhesions on the posteromedial side of the knee, which would ensure that the knee was extended regardless of the hip joint position, we opted for OWHTO using a medial approach. We planned to set the knee at 62.5% MA, that is, the Fujisawa point, and the correction angle was calculated to be 14°. However, due to the large JLCA in this case, the correction angle was reduced by 1°, resulting in a final correction angle of 13°.

Surgical technique

**Figure 3:**
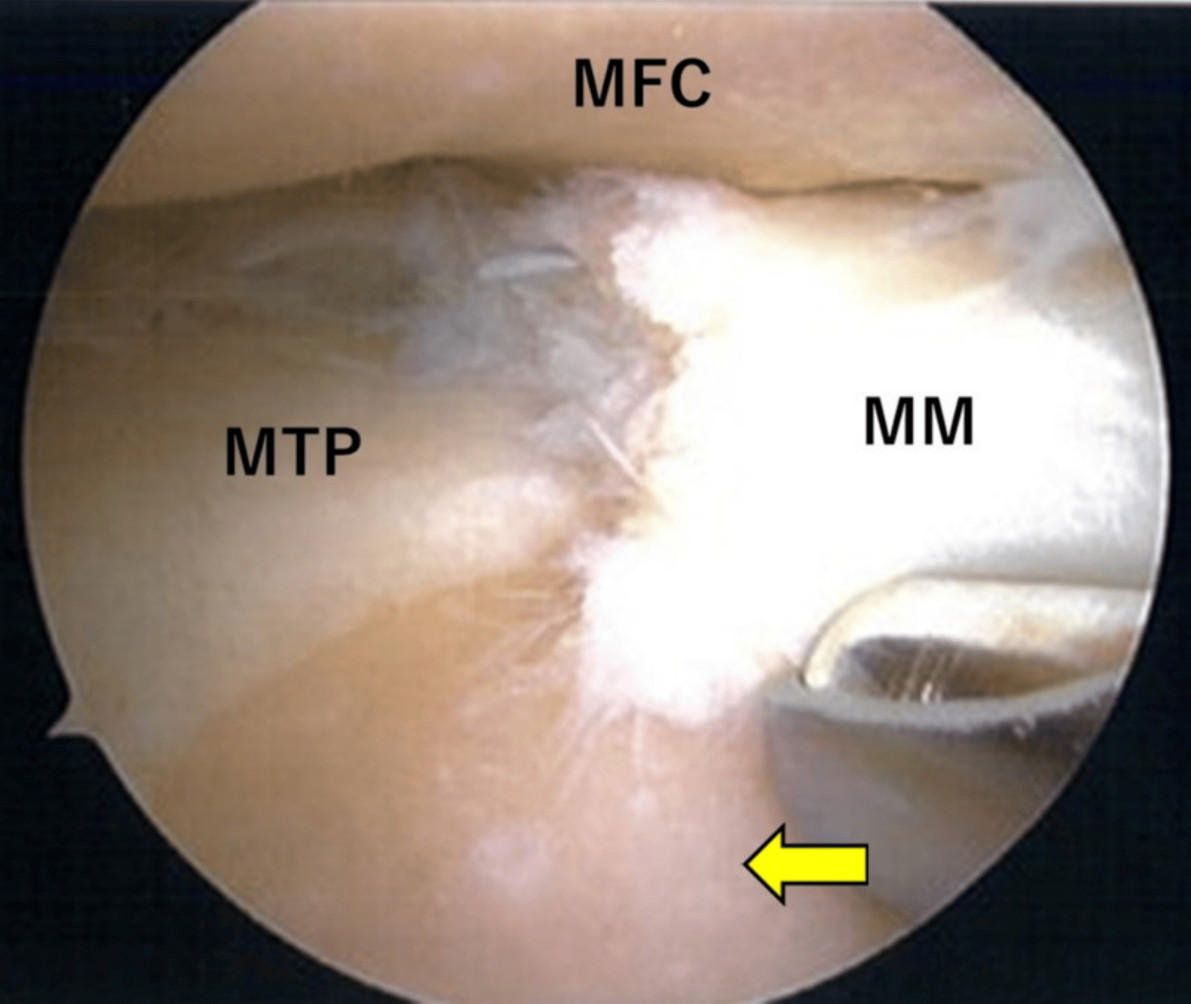
Intraoperative arthroscopy showing degenerative changes in the MM, including tears extending from the midbody to the posterior horn, with ICRS grade 4 defects in both the MFC and MTP (yellow arrow) ICRS, International Cartilage Repair Society; MFC, medial femoral condyle; MM, medial meniscus; MTP, medial tibial plateau

First, we created the anterolateral and anteromedial portals and viewed the interior of the knee joint using an arthroscope. The cartilage of the medial femoral condyle and medial tibial plateau had International Cartilage Repair Society (ICRS) grade 4 defects (Figure 3), whereas that of the lateral femoral condyle and lateral tibial plateau had ICRS grade 1 defects. The cartilage of the PF joint was intact. The medial meniscus showed a degenerative tear from the midbody to the posterior horn with partial resection of the flap injury area. The lateral meniscus remained intact.

A 10-cm skin incision was made medial to the tibia, and the strong adhesions around the semitendinosus and gracilis (STG) tendons were dissected and released. The STG tendon was cut at the tibial insertion, and the medial collateral ligament (MCL) was released. The first oblique osteotomy was performed from the medial side, 3.5 cm distal to the medial tibial plateau and upper proximal tibiofibular joint. Osteotomy was then performed, leaving 5 mm of the lateral cortex (the bone bridge) intact, which served as a hinge point during the opening of the osteotomy. A second frontal osteotomy was performed proximal to the patellar tendon insertion in the first osteotomy plane. The osteotomy site was opened slowly and carefully using special opening equipment to avoid fracturing the lateral cortex. A correction angle of 13° was set under fluoroscopic control, resulting in an opening distance of 11 mm. The size of an artificial bone (Osferion 60®, Olympus Terumo Biomaterials Corp., Shinjuku, Japan) can be easily adjusted during surgery to accommodate the size of the opening using a micro bone saw. An artificial bone graft was placed at the opening of the tibia, the MCL and STG tendons were repaired, and the artificial bone was covered as much as possible. Fixation was achieved using a locking plate (TrisS Green®; TriS Medial HTO Plate System, Olympus Terumo Biomaterials Corp.).

Postoperative therapy

To begin with, the knee ROM training was performed without restrictions. The weight-bearing schedule then progressed gradually. During the first postoperative week, partial weight-bearing (PWB) was set at 10 kg. This increased to one-third of the body weight by the second week and two-thirds by the fourth week. Full weight-bearing was initiated in the fourth week.

Postoperative course

**Figure 4:**
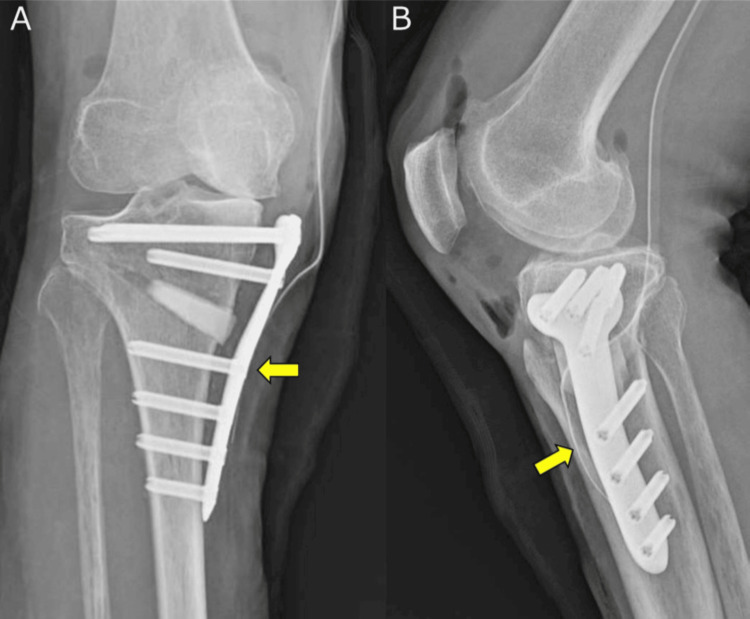
Plain radiograph of the knee immediately after surgery (A) Frontal radiograph not showing an LHF but revealing a distance between the plate and the bone (yellow arrow). (B) Lateral radiograph showing that the plate was placed on the side of the bone (yellow arrow). LHF, lateral hinge fracture

**Figure 5:**
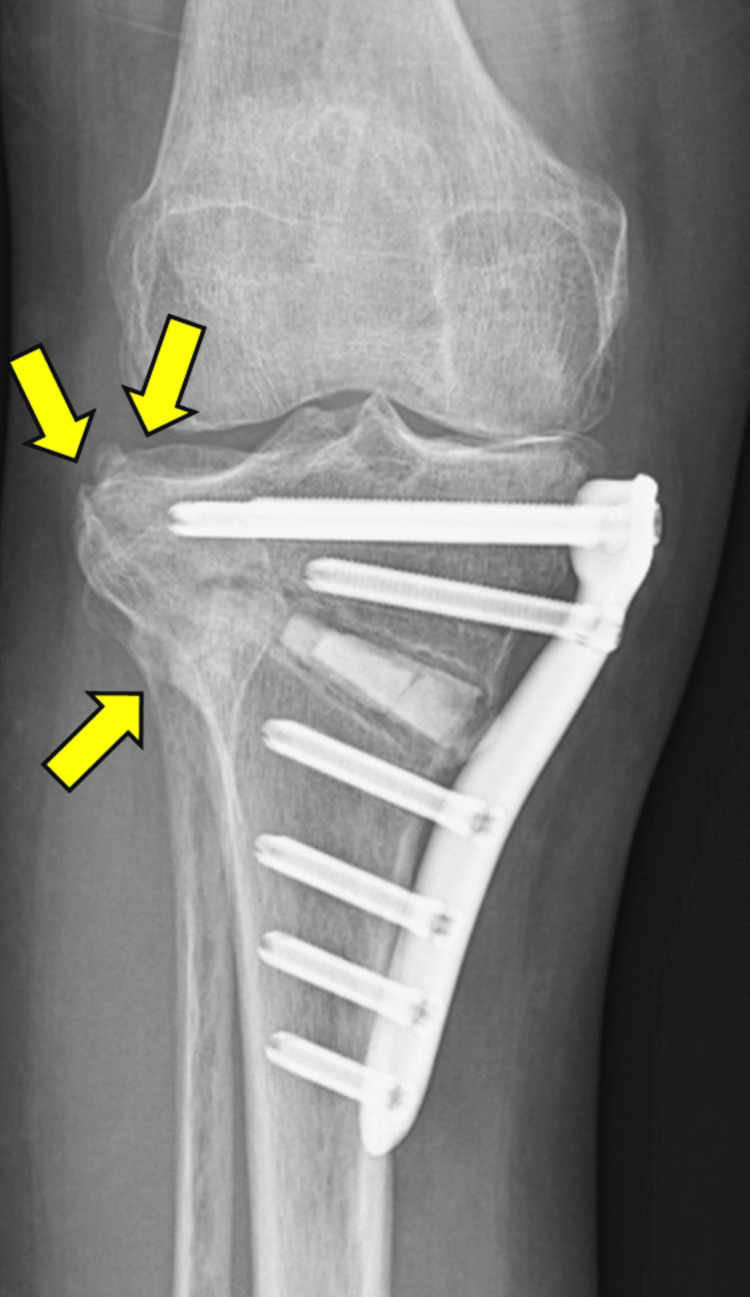
Radiograph taken two months postoperatively showing multiple LHFs (yellow arrows) LHF, lateral hinge fracture

**Figure 6:**
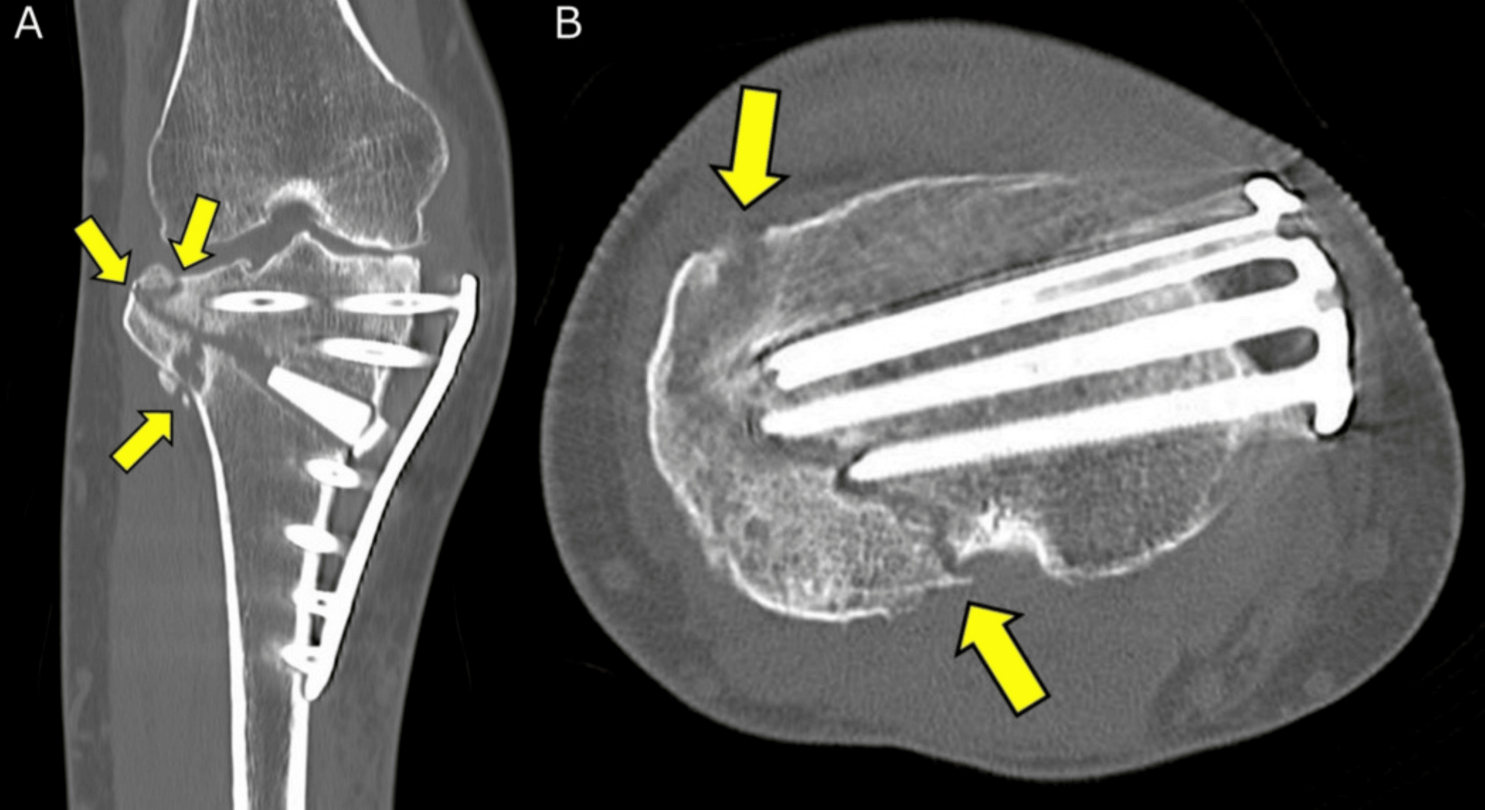
CT image of the knee two months postoperatively (A) Coronal image confirming a combination of types 1, 2, and 3 LHFs (yellow arrows). (B) Axial image showing that the type 3 LHF (yellow arrows) coincides with the tip of the screw, and the bone-plate distance is 10 mm. LHF, lateral hinge fracture

**Figure 7:**
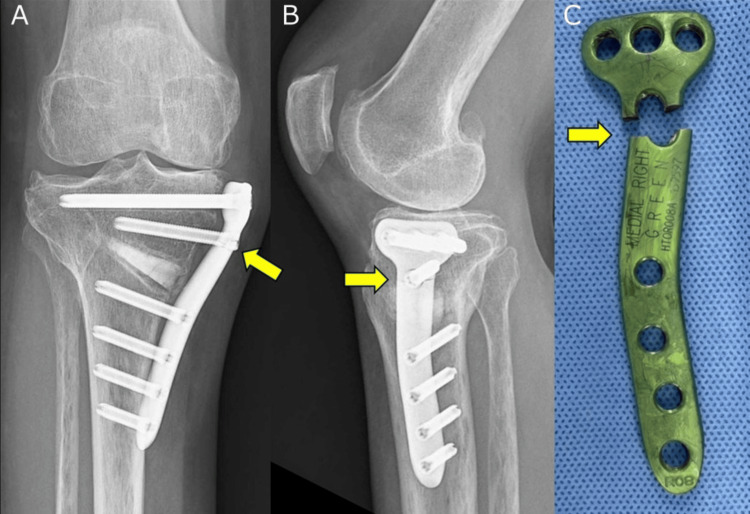
Locking plate showing fracture at the D-hole (A) Frontal and (B) lateral radiographs showing a fracture of the locking plate at 12 months postoperatively (yellow arrow). (C) Fracture of the locking plate at the D-hole observed during its removal at 13 months postoperatively (yellow arrow).

**Figure 8:**
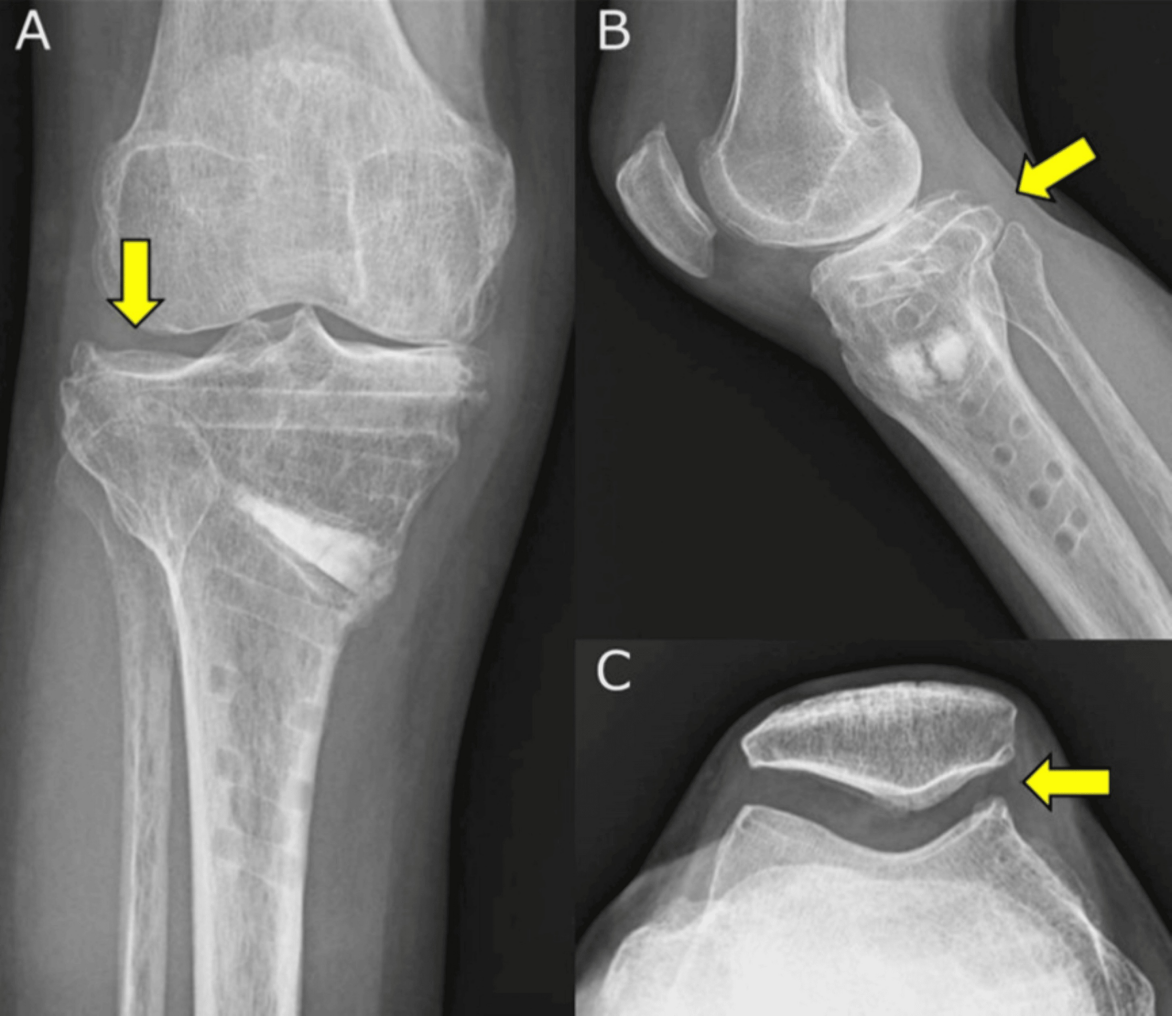
Plain radiograph of the knee at 16 months postoperatively (A) Frontal radiograph revealing a deformity in the lateral tibial joint surface due to the type 3 LHF (yellow arrow). (B) Lateral radiograph showing that the posterior tibial slope has not increased (yellow arrow). (C) Skyline view plain radiograph of the knee at 16 months postoperatively showing no progression of deformation in the PF joint (yellow arrow). LHF, lateral hinge fracture; PF, patellofemoral

**Figure 9:**
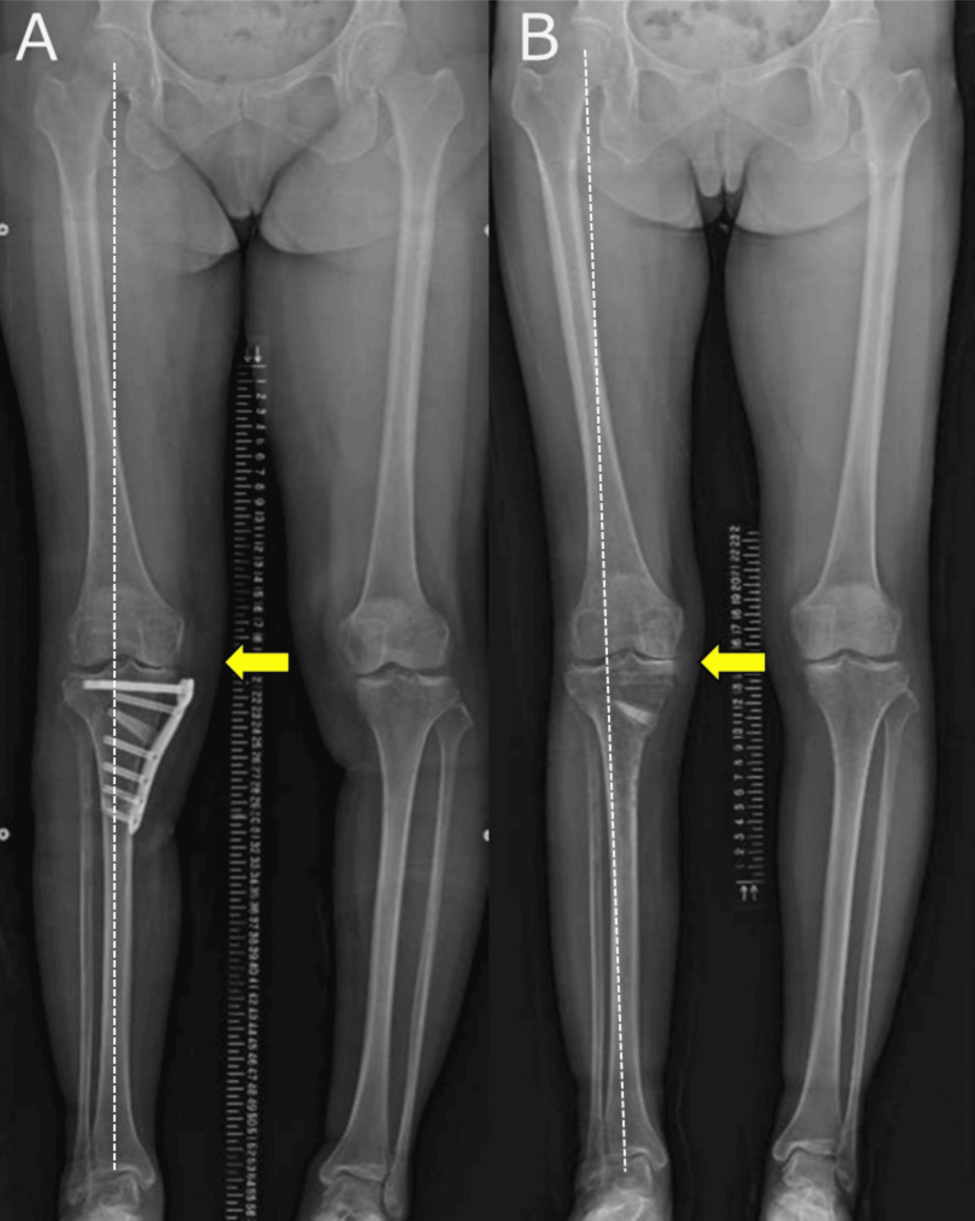
Full-length lower extremity radiograph (A) Radiograph at two weeks postoperatively (yellow arrow). (B) Radiograph obtained at 16 months postoperatively showing bone healing without significant loss of correction (yellow arrow). The white dotted line indicates the Mikulicz line.

No LHF was observed on knee radiographs taken immediately after surgery. Although there was a distance between the plate and bone, the plate was positioned on the lateral side of the tibia (Figure 4). Three weeks after the surgery, the patient was discharged from the hospital after she was able to walk using crutches. Two months postoperatively, radiography suggested the presence of multiple types of LHFs. (Figure 5). Concurrent types 1, 2, and 3 LHFs were observed on coronal CT images. Axial CT images revealed that the type 3 LHF corresponded to the tip of the screw, and the bone-plate distance was 10 mm (Figure 6). During the clinical course, no worsening of the knee pain was observed, although mild tenderness was noted in the lateral hinge area. PWB was advised, and low-intensity pulsed ultrasound (LIPUS) therapy was initiated. Five months postoperatively, there was no worsening of knee pain, and bone union was confirmed. The patient was permitted to walk without assistive devices, and LIPUS therapy was terminated. Due to discomfort caused by the locking plate, removal surgery was performed at 13 months postoperatively. At that time, a plate fracture at the D-hole was observed (Figure 7). A retrospective review of the radiograph revealed that the fracture occurred around 12 months postoperatively. At the 16-month follow-up, the patient was pain-free and could walk smoothly. The patellar ballottement test result was negative, and the knee ROM ranged from 3° to 135°. Abnormal knee flexion caused by hip flexion was resolved. There was no tenderness in the medial or lateral compartments of the knee joint at 16 months postoperatively. Radiography at 16 months postoperatively revealed a deformity of the lateral tibial articular surface due to LHF type 3 and no progression of the deformity of the PF joint (Figure 8). On a full-length lower extremity radiograph, the %MA, mHKA, aFTA, MPTA, and JLCA at two weeks and 16 months postoperatively were 60.5% and 61.3%, −2.6° and −3.3°, 173.3° and 172.4°, 93.7° and 93.9°, and 4.1° and 4.5°, respectively (Figure 9). In the lateral view, the PTS immediately after surgery and 16 months postoperatively were 7.2° and 6.4°, respectively. Bone healing was achieved without significant loss of correction.

## Discussion

We performed OWHTO on a 48-year-old female with a history of lower limb surgery and radiation therapy. Two months postoperatively, the patient had type 1, 2, and 3 LHFs. After treatment with weight-bearing restrictions and LIPUS for three months, bone union was achieved, and no significant correction loss occurred. Sixteen months after the surgery, the patient could walk without difficulty. LHFs, a serious OWHTO complication, are classified into types 1, 2, and 3, which often make treatment challenging for orthopedic surgeons [3]. In clinical reports of LHFs, no correction loss was observed in LHF types 1 and 3, whereas one report noted a maximum correction loss of 7° in LHF type 2 [3]. Additionally, LHF types 1 and 2 show delayed union beyond 24 weeks without correction loss, whereas LHF type 3 shows delayed union beyond 24 weeks with correction loss and joint surface depression (exceeding 2 mm), as noted in a previous study [9]. In the biomechanical evaluation of different types of LHFs in OWHTO, type 3 LHF had the largest wedge displacement compared to types 1 and 2 LHF. On average, the failure loads were significantly reduced in type 3 LHFs compared to those with an intact hinge and those with type 1 LHFs [10]. However, there have been no reports on the combined occurrence of types 1, 2, and 3, and the clinical outcomes of multiple types of LHF remain unclear. Our report describes the clinical course and treatment strategies for multiple concurrent types of LHFs.

The risk factors for LHFs can be categorized into patient- and surgical technique-related factors. The patient-related risk factors include advanced age, obesity, and a large preoperative HKA angle [11]. Atrophic bones become sclerotic and poorly vascularized. Therefore, treating fractures in severely atrophied bones carries a higher risk of losing hardware or nonunion [12]. In this case, the patient was treated for a desmoid tumor and had received radiation therapy, had prolonged pain, and had postoperative disuse, all of which contributed to proximal tibial bone atrophy. Surgical technique-related factors include excessive opening distance [4,5,13], improper hinge point placement either proximal or distal to the tibiofibular joint [14], insufficient osteotomy leading to residual anterior or posterior cortex [15], and the absence of substitute material at the osteotomy site [16]. In an OWHTO cadaver study, there was no significant difference in the failure load between a group with a proximal screw length of 90% without gap filling and one with a proximal screw length of 90% with gap filling. However, the group with a short proximal screw length of 55% without gap filling showed a significantly lower failure load and more lateral cortical bone fractures than the group with a proximal screw length of 90% and gap filling [17]. In a probabilistic-based computational model used to predict the probability of delayed healing or nonunion under different mechanical conditions during fracture treatment for fractures stabilized by locking plates, there was a strong positive linear correlation between the mechanical stimulations in the fracture gap and the distance between the bone and plate [18]. Therefore, we believe that the combined type 1, 2, and 3 LHFs in this patient were multifactorial, owing to a large preoperative HKA angle of 9.5°, proximal tibial bone atrophy, insufficient proximal screw length, and a distance of 10 mm between the bone and the plate.

The treatment for cases involving LHFs includes weight-bearing restrictions [3,9], LIPUS therapy [3,19,20], and the insertion of additional screws or locking plates [9]. Previous studies have shown that the radiolucent zone after OWHTO disappears two months after initiating LIPUS stimulation [3]. In a study in which simultaneous bilateral OWHTO was performed in a single patient with equivalent grades of OA in both knees and comparable varus deformities, LIPUS was applied randomly to one knee, while the other knee served as the control. After four weeks, the increase in callus bone mineral density was significantly greater in LIPUS-treated tibia than in the control tibia [19]. In our case, bone union was achieved without a significant loss of correction through weight-bearing restrictions and LIPUS therapy for three months. Weight-bearing restriction and LIPUS are effective treatments for unstable LHFs.

In future cases involving similar risks, meticulous attention must be paid to both the surgical technique and postoperative management. In terms of surgical technique, the hinge point should be carefully created at an appropriate location, ensuring minimal distance between the bone and the fixation plate. The plate should be positioned laterally, with secure use of sufficiently long screws. In terms of postoperative management, early confirmation of hinge fractures using CT or MRI is essential for tailoring a cautious rehabilitation protocol. In addition, the early initiation of LIPUS therapy may promote bone healing and improve clinical outcomes.

## Conclusions

We report a rare case of combined types 1, 2, and 3 LHFs occurring two months after OWHTO. Potential contributing factors included a large preoperative HKA angle, proximal tibial bone atrophy, insufficient proximal screw length, and distance between the bone and plate. Weight-bearing restriction and LIPUS treatment resulted in bone union without significant loss of correction. To prevent LHF, careful consideration of both patient- and surgical technique-related factors is essential.
